# Physiological and Anatomical Mechanisms in Wheat to Cope with Salt Stress Induced by Seawater

**DOI:** 10.3390/plants9020237

**Published:** 2020-02-12

**Authors:** Rania M. A. Nassar, Hedaya A. Kamel, Ahmed E. Ghoniem, Juan José Alarcón, Agnieszka Sekara, Christian Ulrichs, Magdi T. Abdelhamid

**Affiliations:** 1 Agricultural Botany Department, Faculty of Agriculture, Cairo University, Giza 12613, Egypt; rania.nassar@agr.cu.edu.eg (R.M.A.N.); ahmed.mahmoud@agr.cu.edu.eg (A.E.G.); 2 Radioisotopes Department, Atomic Energy Authority, Dokki, Giza 12311, Egypt; hedaya_kamel@yahoo.com; 3 Irrigation Department, CEBAS-CSIC, Campus Universitario de Espinardo, Murcia 30100, Spain; jalarcon@cebas.csic.es; 4 Department of Horticulture, University of Agriculture in Krakow, Krakow 31-425, Poland; 5 Division Urban Plant Ecophysiology, Faculty of Life Sciences, Humboldt-Universität zu Berlin, Lentzeallee, Berlin 14195, Germany; christian.ulrichs@hu-berlin.de; 6 Botany Department, National Research Centre, 33 El Behouth Steet, Dokki, Cairo 12622, Egypt

**Keywords:** ^14^C photoassimilation, anatomical traits, growth, seawater salt stress, *Triticum aestivum*

## Abstract

Two pot experiments were conducted in a greenhouse to examine ^14^C fixation and its distribution in biochemical leaf components, as well as the physiological and anatomical adaptability responses of wheat (*Triticum aestivum* L.) grown with seawater diluted to 0.2, 3.0, 6.0, and 12.0 dS m^−1^. The results showed significant reductions in chlorophyll content, ^14^C fixation (photosynthesis), plant height, main stem diameter, total leaf area per plant, and total dry weight at 3.0, 6.0, and 12.0 dS m^−1^ seawater salt stress. The ^14^C loss was very high at 12.0 ds m^−1^ after 120 h. ^14^C in lipids (ether extract) showed significant changes at 12.0 dS m^−1^ at 96 and 120 h. The findings indicated the leaf and stem anatomical feature change of wheat plants resulting from adaptation to salinity stress. A reduction in the anatomical traits of stem and leaf diameter, wall thickness, diameter of the hollow pith cavity, total number of vascular bundles, number of large and small vascular bundles, bundle length and width, thickness of phloem tissue, and diameter of the metaxylem vessel of wheat plants was found. In conclusion, salt stress induces both anatomical and physiological changes in the stem and leaf cells of wheat, as well as the tissues and organs, and these changes in turn make it possible for the plants to adapt successfully to a saline environment.

## 1. Introduction

Wheat (*Triticum aestivum* L.) is the leading cereal crop globally. Approximately 85% of the human population derives most of their basic calories and protein from wheat [1]. Wheat is grown in rainfed or irrigated tropical and subtropical lands because of its high degree of acclimatization. However, unfavorable environmental conditions significantly decrease yield efficiency [2,3,4]. About 20% of irrigated agriculture areas are vulnerable to soil salinity, which is considered a global hazard for reducing crop productivity [5]. Based on the Food and Agriculture Organization (FAO) Soils Portal [6], soil is considered saline if it has an electrical conductivity (EC) higher than 0.7 dS m^−1^. The FAO has reported that the total saline soil area is 397 million ha globally, while sodic soils cover 434 million ha [6]. Nelson and Mareida [7] have reported that salinization results in reduced productivity of around 12 million ha of irrigated land.

Worldwide, a quantitative and qualitative decline in fresh water resources available for agriculture has been observed. Therefore, the use of lower-quality water supplies for irrigation purposes will inevitably need to be practiced to maintain economically feasible plant production [8,9]. Several countries have embraced the use of marginal irrigation water to solve water shortages [10]. The industrial use of saline water is a problem of growing concern due to the increasing water demands for irrigation and competition between the urban, industrial, and agricultural sectors of the world economy [11,12]. Egypt is located in an arid zone, so the utilization of seawater in this country has been the latest attempt to obtain satisfactory yields of agricultural and horticultural crops, including soybeans [13], maize [14], faba beans [15], and common beans [16]. The use of 10%–20% seawater concentrations has been reported as economically viable in many projects with cash crops. Castillo et al. [17] reported that the use of 20% marine brackish water had often been proclaimed a great success, but 20% instead of 10% or 15% seawater is still not entirely suitable for agriculture, as many crops fail under ionic and/or osmotic stress. In another report, even water with an EC of more than 3 dS m^−1^ remained severely restricted in terms of agricultural use for irrigation [18]. However, salt water with up to 8 dS m^−1^ (6000 mg L^−1^ TDS) is more or less profitable for irrigation in many regions of the world, under extensively variable soil, weather, and growing conditions.

Salinity stress negatively disturbs basic plant metabolic processes, which in turn results in yield reduction affecting agricultural production, mainly in coastal areas. Due to the storage of salt in the soil through tidal flow, agricultural land can become unproductive. High NaCl concentrations in irrigation water or soil cause serious economic losses from dry lands [19,20], because salinity stress reduces the growth and productivity of many crop species [21,22,23,24,25]. Several physiological and biochemical processes let plants cope with excessive salt concentrations in saline soils. It is essential to analyze the anatomical and metabolome-derived characteristics affected by salt stress, like cell membrane depolarization, disturbed ionic balance, osmotic adjustment, nutrient deficiency, toxic accumulation of Na^+^ in tissues, and the physiological and chemical changes resulting from interactions between multiple stresses [26,27]. Several researchers have focused on anatomical, ecological, physiological and molecular modifications in crops under the stress of salinity [28,29,30]. They found that whole plant metabolism is reprogrammed to increase stress tolerance, which comes with trade-offs and reduced growth rates during the subsequent stages of crops’ development [31].

Salt stress is one of the main constraints of wheat production, and its impact is greater in durum wheat as this type is less salt-stress-tolerant compared to bread wheat. Screening wheat for salt tolerance in seedling and at the maturity stages has been performed [32,33]. However, achieving genetic output increases under salt stress is a challenging task for plant breeders [33]. More research into screening wheat for salt tolerance and proving validity in salt-affected soils is still required to understand the mechanisms involved, and to enhance salt tolerance in this economically crucial crop [34].

Generally, leaf photosynthesis is considered the main source of carbon for plant growth, development, and storage. However, other chlorophyll-containing organs, like the stems or inflorescence components, can make a significant contribution to the general production of photosynthates and influence the carbon partitioning pattern [35]. Carbon partitioning in plants is governed by photosynthesis production, amount, and size of competing sinks, plant organs or tissues, vascular connection, and potential for temporary storage in the leaves. Exposure to ^14^CO_2_ has often been used to explore the photosynthetic efficacy and subsequent motion of photosynthates in various plant species [35].

In this study, we focused on the dependence between the concentration of photosynthetically active pigments, amount of ^14^C lost, and dry matter production, supported by anatomical studies of leaf cross sections of wheat plants under salinity stress. The novel aspect of the present study was in linking the salinity-induced changes in leaf anatomy to the physiological processes which can affect these modifications. We used ^14^C fixation in plants under salinity stress to understand the effect of seawater irrigation on the physiological and anatomical adaptability responses of wheat plants.

## 2. Results

**Figure 1:**
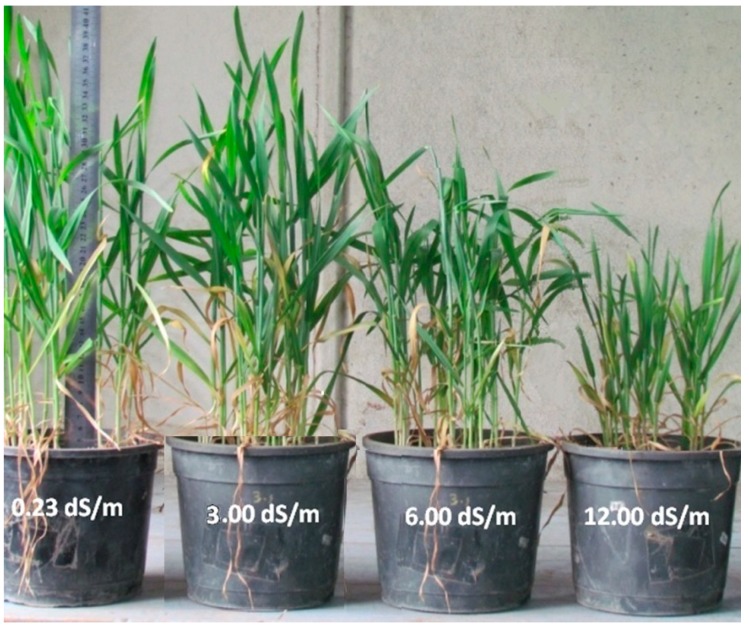
General view of the experiment of wheat plants after subjecting to 0.23, 3.0, 6.0, and 12.0 dS m^−1^ salt stress for 56 days.

**Figure 2:**
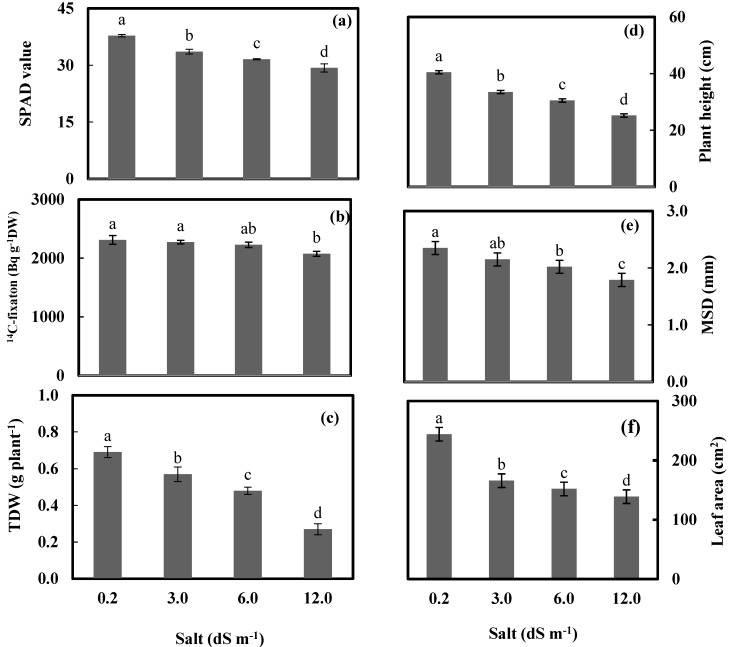
Relative chlorophyll content measured by SPAD meter (SPAD value) (**a**), ^14^C–fixaton in Bq g^−1^ DW (**b**), total dry weight (TDW) (**c**), plant height (**d**), main stem diameter (MSD) (**e**), and leaf area plant^−1^ (**f**) of wheat plants after subjecting at 0.23, 3.0, 6.0, and 12.0 dS m^−1^ salt stress for 56 days. Bars represent standard errors. Different letters along with salt treatments indicate significant differences in access treatment means from 6 replications measured at *p* ≤ 0.05 according to Duncan’s multiple range test.

This research was performed to track the ^14^C fixation and distribution in biochemical leaf components, as well as to analyze the mechanism of acclimation processes of wheat grown for 56 days at 0.2, 3.0, 6.0, and 12.0 dS m^−1^ seawater salt stress at the physiological and anatomical level. A general view of the experimental plants is shown in Figure 1. Figure 2 presents relative chlorophyll content (SPAD value), ^14^C fixation, total dry weight (TDW), plant height, main stem diameter (MSD), and leaf area per plant of wheat after exposure to 0.23, 3.0, 6.0, and 12.0 dS m^−1^ salt stress for 56 days.

### 2.1. Relative Chlorophyll Content

Leaf damage resulting from chlorophyll degradation is a salt-susceptibility screening which can be quickly and simply measured with a portable SPAD meter.

### 2.2. ^14^C Fixation

The reduction in ^14^C fixation was most significant at 12.0 dS m^−1^, reaching 10.2% compared to the control (0.23 dS^−1^) (Figure 2b). Table 1 shows an increase in the amount of ^14^C lost over time from either the control or seawater-irrigated wheat plants. The ^14^C loss was most significant at 12.0 dS m^−1^ after 120 h.

### 2.3. Wheat Growth

It was shown that all levels of salt induced significant decreases in the studied morphological characteristics. The maximum significant decreases were detected at a salt level of 12 dS m^−1^, of 37.0%, 23.8%, and 43.1% for plant height, main stem diameter, and total leaf area per plant, respectively. In addition, the seawater salt stress at 12.0 dS m^−1^ resulted in a sharp decrease of most characteristics under study; for example, plant height and leaf area were recorded at 37.0% and 43.1% below the control. TDW was significantly reduced by seawater application, and the reduction was gradual with an increasing level of salt stress. TDW was decreased by 17.4%, 30.4%, and 60.9% at seawater treatments of 3.0, 6.0, and 12.0 dS m^−1^, respectively, compared to the control. All concentrations of salinity exhibited a significant decrease related to the control. The minimum decrease was at a dose of 3.0 dS m^−1^. In this treatment, plant height, mean stem diameter, and total leaf area presented decreases of 17.5%, 8.5%, and 31.75% below the control, respectively. At 6.0 dS m^−1^ treatment, a significant decrease in the main stem diameter of 23.8% below the control values was found.

### 2.4. Response Curve of Wheat Dry Weight to Seawater Salt Stress

**Figure 3:**
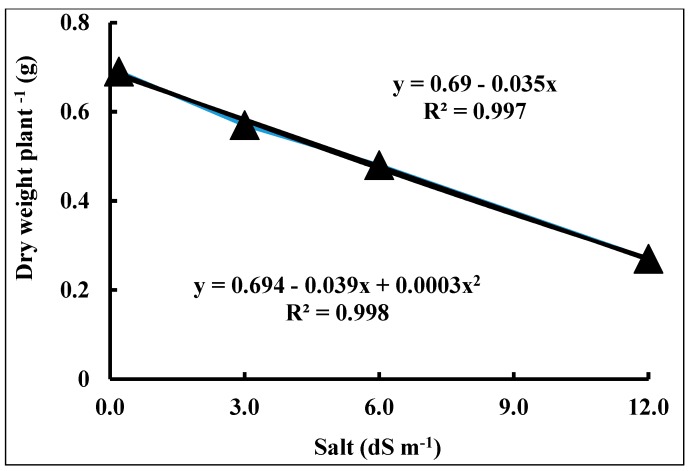
Dry weight of wheat (*Triticum aestivum* L.) cv. Sakha 93 as a function of seawater stress for 56 days.

Figure 3 shows that with a seawater salt stress level increase of 1 dS m^−1^, the TDW was expected to decrease by 0.04 g plant^−1^. A total 99.7% of the variation in TDW was described by the model of linear regression.

### 2.5. Anatomical Studies

**Figure 4:**
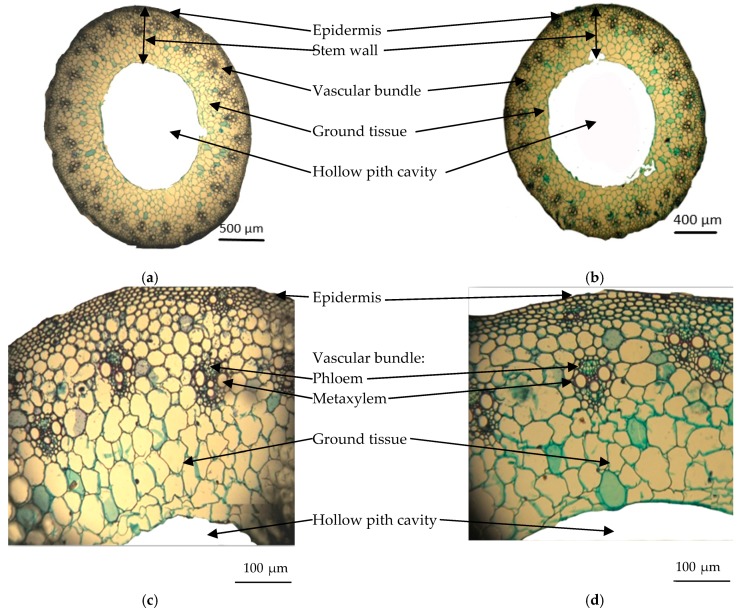
Transverse sections through the median portion of the main stems of wheat plants grown for 56 days at 0.23 and 6.0 dS m^−1^ salt stress (×68) (**a**) 0.23 dS m^−1^; (**b**) 6.0 dS m^−1^, and magnified portion from transverse sections shown in (**a**,**b**) (×330); (**c**) 0.23 dS m^−1^; (**d**) 6.0 dS m^−1^.

It was observed that all concentrations of salinity significantly decreased the anatomical characteristics of wheat leaf and stem, and the reduction gradually coincided with increasing salt concentration (Figure 4, Table 2). This required additional study of the internal structure of wheat plant vegetative organs grown either under control conditions or under salinity stress. Specimens of the median portions of main stems and their corresponding leaves were tested for vegetative growth traits. Specimens were sampled from wheat plants grown for 56 days at 0.23 and 6.0 dS m^−1^ salt stress.

#### 2.5.1. Main Stem Anatomy

Transverse sections of the medium portions of the main wheat stems through the median portion of wheat plants grown at 0.23 and 6.0 dS m^−1^ salt stress for 56 days are shown in Figure 4a,b. In addition, Figure 4c,d shows magnified portions from the transverse sections presented in Figure 4a,b. Table 2 contains the values of histological features in the transverse sections through the median portions of the wheat plants’ main stems. Table 3 and Figure 4f reveal that salinity stress induced a decrease in stem diameter of 15.9% below the control. The reduction in stem diameter under salt stress included a notable decrease in stem wall thickness by 27.5% below the control, and in the diameter of the hollow pith cavity by 20.5% below the control. It was noted that the total number of vascular bundles per cross section decreased to 26% below the control, due to decreases of 25.8% and 26.3% below the control for the number of large and small vascular bundles, respectively. Salinity decreased bundle length, bundle width, and thickness of phloem tissue of large bundles by 4.3%, 6.1%, and 4.3% of the control, respectively. However, such treatment showed no effect on the diameter of the metaxylem vessels of large bundles.

#### 2.5.2. Leaf Anatomy

**Figure 5:**
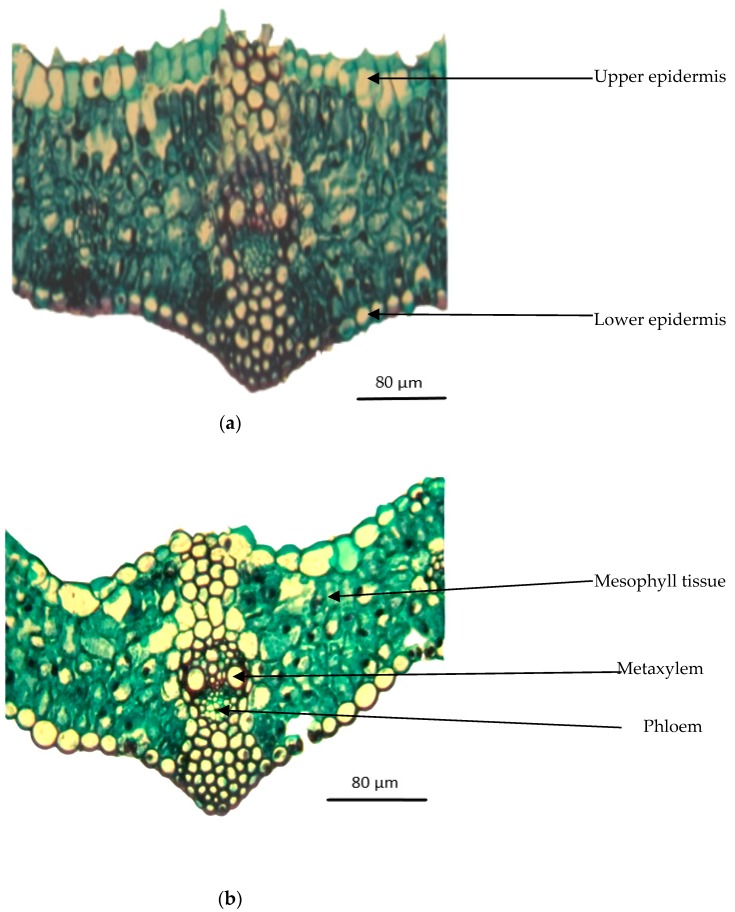
Transverse sections through the median portion of lamina of a leaf developed at the median portion of the main stem of wheat plants grown for 56 days at 0.23 and 6.0 dS m^−1^ salt stress. (×330). (**a**) (0.23 dS m^−1^); (**b**) (6.0 dS m^−1^).

Figure 5a,b shows transverse sections of the medium portion of the leaf lamina developed in the main stem of wheat plants grown at 0.23 and 6.0 dS m^−1^ salt stress for 56 days. Measurements of histological traits are shown in Table 2. Salt stress at 6.0 dS m^−1^ reduced leaf lamina thickness to 4.5% below the control (0.23 dS m^−1^). This reduction could be ascribed to the reduction of the thickness of the mesophyll tissue to 4.5% below the control, and in the dimensions of the main vascular bundle by 5.8% in length and by 14.3% in width. Likewise, thickness of the phloem tissue and diameter of the metaxylem vessel were decreased to 16.4% and 2.0% below the control, respectively.

## 3. Discussion

### 3.1. Chlorophyll Content

The results revealed a significant reduction in the chlorophyll content of wheat plants treated with diluted sea water, compared with the control, at each time interval. Moreover, the reduction was the highest at the highest salt level of 12 dS m^−1^. Similar results have been recorded for other crops in the literature, including for three tomato cultivars by Hajer et al. [36]. Seawater irrigation resulted in a substantial reduction in the content of total chlorophyll, chlorophyll a, and chlorophyll b as compared to the values noted for wheat plants irrigated with fresh water [37]. In the soybean (*Glycine max* L.) cultivar Giza 111, seawater salt stress at 3.13 or 6.25 dS m^−1^, applied for 45 days, decreased chlorophyll a, chlorophyll b, carotenoid, and total pigment concentrations; moreover, the reduction gradually coincided with increased salt stress levels [13]. Chlorophyll content reduction was prevalent under salt stress, and chlorophyll was used as a precise marker for cellular metabolic status in studies by Orabi and Abdelhamid [15]. Reduction of photosynthetic pigment contents under salt stress was reported as the effect of distinct factors, commonly linked to the decay of the cellular membranes [38,39].

### 3.2. ^14^C Fixation

Some reasons for the decrease of photosynthesis due to salinity include that (i) salt stress damages the thylakoid membrane, interferes with its functions, and ultimately reduces photosynthesis and plant yields [40,41], as photosynthetic apparatus conservation is an important strategy for increasing crop yields under stress; (ii) photosynthesis is decreased because of the reduction of the leaf growth rate, leaf area, and leaf duration. Moreover, photosynthesis and respiration per unit of leaf area are also influenced, and (iii) stomatal closure in plants under salt stress is regarded to be one of the primary reasons for the reduction in photosynthesis, because of lower accessibility of CO_2_ in the mesophyll [42]. Mineral uptake is influenced by an imbalance in the accessibility of various ions in salt stress conditions [20]. The present research showed an increase in the amount of ^14^C lost over time from both control and seawater-irrigated wheat plants; the most intensive loss occurred at 12.0 dS m^−1^ after 120 h. The ^14^C lost from leaves may have been an effect of respiration or translocation to other plant organs including buds, stem, or roots. Since both source and sink activities change dynamically with water stress, the distribution of fixed photoassimilates can vary with time. However, the physiological status of source leaves, like the photosynthetic level and sucrose accumulation, could be strongly influenced by drought stress [43], and could have a significant effect on the export rate of newly set carbon [44]. We determined that ^14^C in lipids (ether extract) showed significant changes with 12.0 dS m^−1^ treatment at 96 and 120 h. In ethanol-soluble compounds, ^14^C was highest at 24 h in the control and 3.0 dS m^−1^-treated plants then decreased by 6.0 and 12.0 dS m^−1^ caused fluctuations in the ^14^C during the experimental period. Kamel et al. [35] found the highest amount of ^14^C in an ethanol extract after 24 h in the three genotypes of soybean subjected to water stress.

### 3.3. Growth of Wheat

Salts in the soil water may inhibit plant growth/yield for two reasons: first, the presence of salt in the soil solution reduces the ability of the plant to take up water, leading to a reduction in the growth rate. Second, if excessive amounts of salt enter a plant with the transpiration stream there will be injury to cells in the transpiring leaves, causing further reductions in growth. The synergism of both effects is also possible [45,46]. Such results are in agreement with those reported by several authors on wheat plants [33,47]. In controlled conditions, assessment of salinity tolerance by evaluating morphological characteristics is more viable than in agronomical ecosystems [48]. Dry matter accumulation at an early stage of the wheat growth could be used to differentiate between resistant and susceptible germplasms [49]. Therefore, based on the proportion of biomass under salt treatment/control, the salt tolerance index can be estimated [46,47]. Ashraf et al. [49] indicated that a positive correlation in early seedling phases between dry matter and plant height may be a reliable function for screening salt-stress wheat genotypes. In our study, there was strong linear correlation between plant height and total dry weight (TDW) (*r* = 0.97; *p* ≤ 0.01). The decrease in photosynthetic pigments and photosynthetic capacity might have been the main reason for the TDW reduction of wheat irrigated with saline water. It has been stated that salinity stress severely restricts the growth and productivity of winter wheat [48,50]. Moreover, it was determined in the present study that a lower salinity level was followed by a decrease in most traits, which was in line with the other reports showing that an increasing NaCl level reduces the shoot length and biomass of wheat seedlings [51]. Tammam et al. [52] noticed that application of saline water with 0, 60, 120, 240, and 320 mM NaCl resulted in a reduction in the leaf area of wheat, and a significant decrease was obtained under 240 and 320 mM NaCl. In another study, Baum et al. [53] reported that a reduction in water flow was mainly due to the decrease in the salt-induced growth rate of sorghum and the related decline in leaf area. Additionally, Abd Elbar et al. [54] showed progressive reduction in plant height and fresh weight with increasing salinity levels of 100, 200, and 300 mM NaCl in *Leptochloa fusca* L. Kunth plants.

### 3.4. Anatomical Studies

Most plant physiological processes associated with salinity are linked to anatomical structure adaptation, which allows plants to grow under abiotic stress [55]. For instance, salt stress resulted in physiological and morpho-anatomic changes in *Lotus tenuis* [56]. The obtained results on wheat plants grown under salt stress in terms of the anatomical structures of the main stems and leaves are supported by previous reports on wheat [32], kallar grass [54], and faba beans [16,29]. Moderate and high salinity concentrations (3000 and 6000 mg kg^−1^ NaCl) reduced xylem and phloem tissue and metaxylem vessel diameter, as well as the primary sorghum bundle size [57]. In the present study, seawater salt stress decreased the cross-sectional area of the vascular bundle throughout the stems and leaflets, resulting in a significantly diminished conductive potential of the phloem and xylem. Moreover, seawater application reduced the vascular bundle area and the vessel diameter. Salt stress increased the flowing resistance of water from roots to leaves, reduced vascular tissue transportation efficiency, and restricted the transportation of water due to dissolved salt ions absorbed by the roots [32]. However, seawater salt stress had a more visible effect on phloem than on xylem, in which translocation of water dissolved salt ions was severely restricted to the ground parts, and the transportation of photosynthetic materials was decreased to the plant apex and young roots.

## 4. Materials and Methods

### 4.1. Experimental Procedures

Two pot experiments were conducted in a greenhouse of the Radioisotope Department, Nuclear Research Center, Dokki, Cairo, Egypt (30°02′09.5″ N 31°12′18.5″ E), during the winter season of 2013/14. The aim of the research was to investigate ^14^C fixation and distribution in biochemical leaf components, and to track the physiological and anatomical adaptability responses of wheat irrigated with seawater in different dilutions.

During the experiments, daily temperature ranged from 12.3 to 17.0 °C with an average of 15.4 ± 1.0 °C. The average night temperature was 11.1 ± 1.6 °C, with a minimum and maximum of 7.9 and 15.5 °C, respectively, while average day temperature was 22.0 ± 2.2 °C, with a minimum and maximum of 16.8 and 27.3 °C, respectively. Daily relative humidity ranged between 21.0% and 57.0% with a mean value of 48.6% ± 8.1%.

The experiments were established in a completely randomized design with four diluted seawater salt stress treatments replicated six times. Diluted seawater water was prepared by mixing fresh water of 0.23 dS m^−1^ with seawater of 51.2 dS m^−1^ to achieve the required salt levels of 0.2, 3.0, 6.0, or 12.0 dS m^−1^. Each experimental treatment consisted of 24 pots.

The investigated material was wheat (*Triticum aestivum* L.) cv. Sakha 93. Grains of uniform size and color were washed with distilled water, sterilized with 10% sodium hypochlorite solution for about 15 min, washed again with distilled water, and air-dried. The grains were then sown in plastic pots (Ø 16 cm) filled with 2 kg of sandy soil from the Experimental Farm of the National Research Centre, Nubaria, Egypt. Ten grains were sown per pot, at 30 mm depth. The characteristics of soil and irrigation water used in the two pot experiments are presented in Table 3. The mineral fertilizers were mixed with the soil three days before cultivation in the following doses: (1) ammonium sulfate (20.5% N) at a dose of 800 kg ha^−1^; (2) super phosphate (15% P_2_O_5_) at a dose of 240 kg ha^−1^; and (3) potassium sulfate (48% K_2_O) at a dose of 120 kg ha^−1^. All agricultural practices for wheat production were conducted according to the recommendations of the Egyptian Ministry of Agriculture and Land Reclamation.

The first experiment was laid out in a completely randomized design with four seawater salt stress treatments (0.2, 3.0, 6.0, or 12.0 dS m^−1^) replicated six times, to investigate ^14^C fixation and its distribution in biochemical leaf components. In the first experiment, wheat grains were sown on November 22, 2013. The second experiment was laid out in a completely randomized design with four seawater salt stress treatments (0.2, 3.0, 6.0, or 12.0 dS m^−1^) with six replications to examine the physiological and anatomical responses of wheat plants. In the second experiment, wheat grains were sown on November 22, 2013 and harvested on January 31, 2014. Each experiment consisted of 24 pots.

In both experiments, wheat seedlings were thinned 10 days after sowing, and four uniform seedlings per pot were left for future investigations. Seedlings were irrigated with equal volumes of tap water until 15 days after sowing. Starting from the 16th day, all pots were irrigated with either tap water (0.23 dS m^−1^) or different dilutions of seawater (3.0, 6.0 or 12.0 dS m^−1^) until harvest. Diluted seawater was prepared by mixing fresh water of 0.23 dS m^−1^ with seawater of 51.2 dS m^−1^ to achieve the required seawater salt levels of 3.0, 6.0, or 12.0 dS m^−1^.

Soil water field capacity, namely 0.15, was estimated by saturating the soil in the pots with water, draining for 48 h, and weighing. Soil water capacity was maintained at about 90% of the maximum water capacity by weighing the pots and balancing the daily loss of water.

### 4.2. ^14^CO_2_ Fixation and Distribution Analysis

To measure ^14^CO_2_ fixation, on the 21st day after inducing salt treatment, the pots were placed in a glass chamber. As a result of 10% HCl and NaH_14_CO_3_ reaction, ^14^CO_2_ was generated. NaH_14_CO_3_ with an original specific activity of 23.2 MBq mg^−1^ (Radiochemical Laboratory, Ameresham, UK) was used as a reagent. After 15 min of exposure, excess ^14^CO_2_ was trapped in a 1N NaOH. The pots were transferred to a greenhouse. Plant leaves were harvested at the end of ^14^CO_2_ exposure, then at 24, 48, 96, and 120 h after ^14^CO_2_ exposure. Plant material was frozen for 30 min, then oven-dried and used in the measurements [35]. Dried samples, collected at the end of ^14^CO_2_ exposure, were combusted using a Harvey Biological Oxidizer (OX–600, Hillsdale, NJ, USA). The ^14^CO_2_ was trapped in a carbosorb scintillation cocktail and counted in a TRI-CARB 2300 Liquid Scintillation Analyzer (Packard, Dreieich, Germany) [35]. To measure the loss of ^14^C from leaves, the dried samples collected at 24, 48, 96, and 120 h after exposure to ^14^CO_2_ were combusted and counted as mentioned above, and the ^14^C which disappeared from the leaves was calculated according to Kamel et al. [35]. Oils were extracted in petroleum ether at a temperature of 60–80 °C via the Soxhlet method (A.O.A.C. 1990). Oil-free tissues were extracted with 80% ethanol (v/v) [58]. The homogenates were then centrifuged using a MPW-351 R centrifuge (MPW.MED. INSTRUMENTS, Warsaw, Poland) with rcf 4226 for 10 min. The ethanol extract was evaporated and ^14^C was measured using a liquid scintillation analyzer.

### 4.3. Morphological Measurements, Chlorophyll Content, and Dry Weight of Wheat

The chlorophyll of wheat leaves was measured using a hand-held battery portable optical meter (Minolta SPAD-502Plus chlorophyll meter, Tokyo, Japan), which records the absorbance of the leaf in two regions, red (650 nm) and infrared (940 nm). Index values (SPAD value) were used to specify the relative leaf chlorophyll content, but not the absolute chlorophyll content or concentration. The third fully expanded leaf per plant was used for measurements, which were performed at six positions along the length and averaged. The SPAD meter sensor fully covered the leaf blade, avoiding interference from the veins. Measurements of chlorophyll were performed at 56 days after inducing salt stress.

Wheat plants were sampled at 56 days from inducing seawater salt stress for anatomical studies and measurements of morphological parameters, including the plant height, main stem diameter, and total leaf area per plant. Moreover, sampled plants were oven-dried for 72 h at 70 °C, and the dry weight of each plant was recorded.

### 4.4. Anatomical Studies

To determine the anatomical responses, the main stems and leaves of wheat plants grown for 56 days at 0.23 and 6.0 dS m^−1^ salt stress were used for preparation of transverse sections. Specimens were fixed for 48 h in FAA (10 mL formalin, 5 mL glacial acetic acid, 50 mL ethyl alcohol 95%, and 35 mL distilled water). The samples were then washed in 50% ethyl alcohol, dehydrated in a normal butyl alcohol series, embedded in paraffin wax with a melting point of 56 °C, sectioned to a thickness of 20 µm, double-stained with safranine-light green, cleared in xylem, and mounted in Canda Balsam [59,60]. Six sections per treatment were prepared, observed, and photomicrographed with a microscope (AxioPlan, Zeiss, Jena, Germany).

### 4.5. Statistical Analysis

All data were subjected to an analysis of variance (ANOVA) for a completely randomized design [61], after testing for the homogeneity of error variances using Levene’s test [62] and testing for normality distribution according to Shapiro and Wilk [63]. Statistical analysis was performed using COSTAT computer software (CoHort Software version 6.303, Berkeley, CA, USA). Statistically significant differences between means were compared at *p* ≤ 0.05 using Duncan’s multiple range test.

## 5. Conclusions

A significant decrease occurred in chlorophyll content, ^14^C fixation (photosynthesis), plant height, main stem diameter, total leaf area per plant, and total dry weight after 3.0, 6.0, and 12.0 dS m^−1^ seawater treatment (salt stress) applied to wheat plants. The decrease in all mentioned traits was accompanied by a reduction in the anatomical characteristics of the stem and leaf, including the diameter, wall thickness, diameter of the hollow pith cavity, total number of vascular bundles, number of large and small vascular bundles, bundle length and width, thickness of phloem tissue, and diameter of the metaxylem vessel of the wheat plants. Such adaptive wheat traits can be used to classify salt tolerance genotypes in wheat and other plant species in salt-stressed conditions. Using current molecular and genetic engineering methods, the anatomical adaptation features could also be targets for integration into salt-susceptible species. In conclusion, salt stress induced structural alterations in the cells, tissues, and organs of wheat, and these modifications in turn allowed for a successful adaptation to a saline environment.

## Figures and Tables

**Table 1:** Distribution of ^14^C (Bq g^−1^ DW) in ethanol-soluble compound and ether extract (lipid) of wheat plant leaves at 0, 24, 48, 96, and 120 h after subjecting wheat plants to 0.23, 3.0, 6.0, and 12.0 dS m^−1^ salt stress for 21 days^#^.

Time (h)	^14^C	Seawater Salt Stress (dS m^−1^)			
		0.2	3.0	6.0	12.0
0	Total	2310 ± 40.1 a^†^	2273 ± 33.3 a	2227 ± 35.2 ab	207 ± 28.5 b
	Lost (respired)	0.00			
	Ethanol extract	591 ± 30.2 a	567 ± 28.4 a	461 ± 17.0 b	598 ± 13.1 a
	Ether extract	34 ± 2.84 a–c	32 ± 2.40 bc	40 ± 1.45 a	27 ± 2.00 c
24	Total	2281 ± 52.5 a	2206 ± 61.2 ab	2135 ± 20.2 a-c	1989 ± 39.3 c
	Lost (respired)	29	67	92	85
	Ethanol extract	680 ± 09.2 a	571 ± 07.3 b	441 ± 10.4 d	585 ± 08.4 b
	Ether extract	39 ± 4.97 a	40 ± 3.78 a	30 ± 1.76 a	37 ± 2.96 a
48	Total	2129 ± 39.8 a	2113 ± 19.1 a	2043 ± 40.4 ab	1892 ± 53.1 b
	Lost (respired)	181	160	184	182
	Ethanol extract	506 ± 03.2 a	507 ± 05.5 b	443 ± 12.3 c	611 ± 14.9 a
	Ether extract	35 ± 4.09 a	39 ± 1.76 a	37 ± 3.84 a	37 ± 2.91 a
96	Total	2003 ± 13.3 a	1991 ± 43.9 a	1946 ± 36.9 a	1720 ± 17.3 b
	Lost (respired)	307	282	281	354
	Ethanol extract	519 ± 04.8 a	449 ± 01.5 b	541 ± 20.7 a	554 ± 09.6 a
	Ether extract	46 ± 5.49 a	28 ± 1.00 c	49 ± 1.76 a	37 ± 0.88 b
120	Total	1951 ± 35.2 a	1926 ± 32.3 a	1895 ± 60.6 a	1560 ± 67.6 b
	Lost (respired)	359	347	332	514
	Ethanol extract	399 ± 01.5 d	557 ± 21.3 b	602 ± 12.7 a	515 ± 05.9 c
	Ether extract	35 ± 0.67 ab	39 ± 0.88 a	30 ± 3.50 b	39 ± 1.20 a

^‘†^ Mean values in the same row for each trait followed by the same lower-case letter are not significantly different according to Duncan’s multiple range test at *p* ≤ 0.05. ^#^ Measurements were made at 0, 24, 48, 96, 120 h after subjecting wheat plants at 0.2, 3.0, 6.0, and 12.0 dS m^−1^ salt stress for 21 days.

**Table 2:** Histological characteristics of transverse sections of median portion of the main stems and leaves of wheat plants after subjecting to 0.23 and 6.0 dS m^−1^ salt stress for 56 days.

Histological Characters	Salt Stress (dS m^−1^)		± Salt Stress 0.23 dS m^−1^ (%)
	0.23	6.00	
**Stem:**			
Stem diameter (µm)	2460.0^†^	2070.0	−15.9
Stem wall thickness (µm)	600.0	435.0	−27.5
Diameter of hollow pith cavity (µm)	1320.0	1050.0	−20.5
Total No. of vascular bundles	50.0	37.0	−26.0
No. of large vascular bundles	31.0	23.0	−25.8
No. of small vascular bundles	19.0	14.0	−26.3
Histological characters of large bundle:			
Length (µm)	117.0	112.0	−4.3
Width (µm)	89.5	84.0	−6.1
Thickness of phloem tissue (µm)	35.0	33.5	−4.3
Diameter of metaxylem vessel (µm)	33.5	33.5	=
**Leaf:**			
Thickness of blade (µm)	193.5	179.0	−4.5
Thickness of mesophyll tissue (µm)	165.5	158.0	−4.5
Histological characters of large bundle:			
Length (µm)	86.5	81.5	−5.8
Width (µm)	84.0	72.0	−14.3
Thickness of phloem tissue (µm)	36.5	30.5	−16.4
Diameter of metaxylem vessel (µm)	25.5	25.0	−2.0

^†^ Means of three sections from three specimens.

**Table 3:** Electrical conductivity (EC), pH, cations, and anions of irrigation water and soil used in the pot experiments.

	EC	pH	Cations (mmol L^−1^)				Anions (mmol L^−1^)			
	dS m^−1^		Ca^2+^	Mg^2+^	Na^+^	K^+^	HCO_3_^−^	CO_3_^2−^	SO_4_^2−^	Cl^−^
Water:										
Tapwater	0.23	7.35	0.50	0.25	2.40	0.20	0.10	0.00	0.65	2.70
Seawater	51.2	7.76	21.60	7.56	454.57	1.51	6.05	0.00	38.18	432.00
Soil:										
Sandy	0.14	8.11	1.15	1.10	1.31	0.24	1.13	0.00	2.11	0.70
